# Parietal Thoracic Tuberculosis: A Report of Two Cases

**DOI:** 10.7759/cureus.56770

**Published:** 2024-03-23

**Authors:** Oussama Afandi, Amal Miqdadi, Nouhad Benmansour, Benslima Najwa

**Affiliations:** 1 Thoracic Surgery, Cheikh Khalifa International University Hospital, Mohammed VI University of Health Sciences, Casablanca, MAR; 2 Nuclear Medicine, Cheikh Khalifa International University Hospital, Mohammed VI University of Health Sciences, Casablanca, MAR; 3 Neurological Surgery, Cheikh Khalifa International University Hospital, Mohammed VI University of Health Sciences, Casablanca, MAR; 4 Radiology, Cheikh Khalifa International University Hospital, Mohammed VI University of Health Sciences, Casablanca, MAR

**Keywords:** chest ct scan, thoracic surgery, postpartum tuberculosis, extrapulmonary tuberculosis (eptb), chest wall mass

## Abstract

Parietal thoracic tuberculosis is a rare localization of tuberculosis. It reaches the ribs and intercostal spaces due to hematogenous spread or direct transcutaneous inoculation. The diagnosis of tuberculosis must be evoked given the endemic context, even in immunocompetent patients. Surgical excision associated with medical treatment remains the best treatment in this case to avoid any local or distant recurrence.

## Introduction

Tuberculosis remains an endemic disease in Morocco, but parietal localization remains a rare extrapulmonary form, representing 15% of extrapulmonary tuberculosis cases and only 0.1% of tuberculosis localizations [1]. It presents as a collection of abscessed tissue masses. The diagnosis is determined by clinical signs and radiology and confirmed by bacteriology and/or histology [2]. Medical treatment alone is often insufficient and must be accompanied by surgery. Here, we report the case of two patients.

## Case presentation

Observation 1

**Figure 1:**
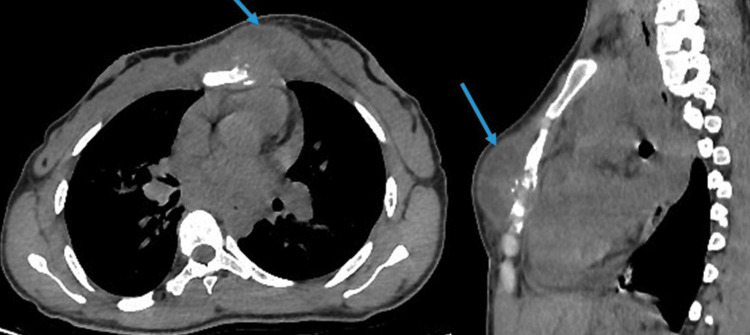
Axial (A) and sagittal (B) sections of the chest CT scan Images showing a left-sided presternal mass (blue arrow) with osteolytic lesion in the sternum (arrow)

**Figure 2:**
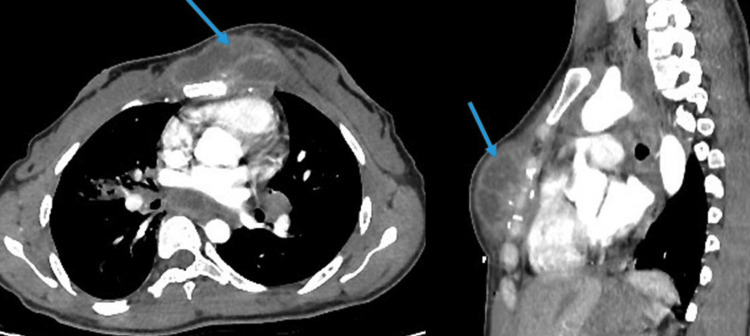
Axial (A) and sagittal (B) sections of the contrast-enhanced CT scan Images showing a left-sided presternal mass (blue arrow) with calcification

The patient was a 22-year-old woman with no medical history who consulted us for remittent presternal swelling and dyspnea. On questioning, tuberculosis contagion was not suspected. Physical examination indicated a large, hard, fixed, left-middle presternal mass with visible signs of fistulization and inflammation. No nipple discharge or swelling of the breasts was observed. The rest of the clinical examination results were normal. A chest CT scan indicated a cold abscess associated with mediastinal adenopathy with sternal lysis (Figure 1, Figure 2).

The biological assessment showed normocytic normochromic anemia with a red blood cell concentration of 11.2 g/dl with no lymphopenia. Surgery with anatomopathological examination was requested; this confirmed the presence of diffuse epithelioid-gigantocellular granulomatous inflammation, necrosis, and fistulization in the soft tissues. The diagnosis was confirmed by the detection of *Mycobacterium tuberculosis* DNA using GeneXpert. The patient was given anti-tuberculosis treatment for nine months according to the 2RHZE/7RH regime, that is, two months of rifampin/isoniazid/pyrazinamide/ethambutol followed by seven months of rifampicin/isoniazid.

Observation 2

**Figure 3:**
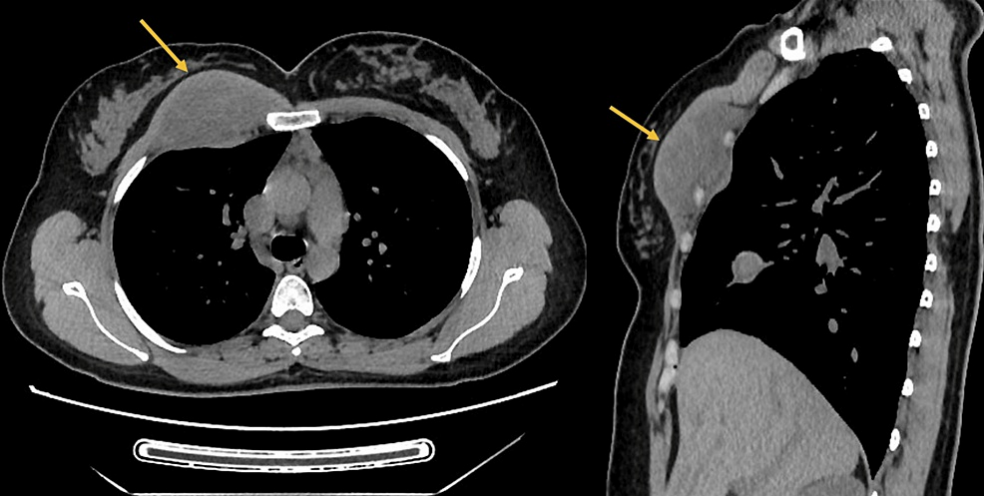
Axial (A) and sagittal (B) sections of the chest CT scan Images showing a right anterior chest wall mass (yellow arrow) infiltrating the parietal pleura

**Figure 4:**
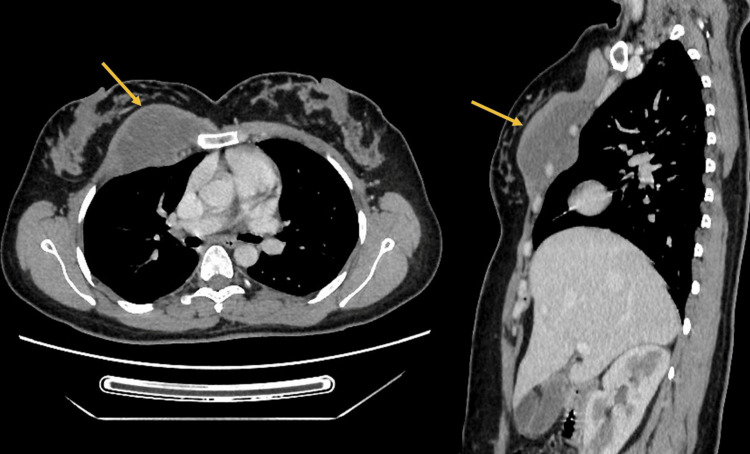
Axial (A) and sagittal (B) sections of the post-contrast chest CT scan Images showing the enhancement of the anterior chest wall mass (yellow arrow)

The patient was a 33-year-old woman who regularly consumed unpasteurized milk. She had had two gestations and two live births. Her medical did not lead us to suspect tuberculosis contagion. She consulted us for a tingling sensation and auto-palpation of a right parietal mass. She did not report night sweats. The general symptoms and apyrexia remained throughout the examination. Physical examination revealed a large, hard, fixed, cold, right parietal mass with no visible signs of fistulization or inflammation. No nipple discharge or swelling of the right breast was observed. The rest of the clinical examination results were normal. A thoracic CT scan identified a thoracic parietal mass on the right pectoral muscle with two components: fluid and tissue (Figure 3, Figure 4). This mass was enhanced after the injection of contrast product and measured 8.5 cm along the longitudinal axis and 5 cm along the anteroposterior axis. However, the mass had infiltrated the anterior pleura without invading the parenchyma or the opposite ribs. In addition, several micronodules were observed, the largest of which measured 7.5 mm.

Biological tests only indicated lymphopenia at 1250 elements/mm^3^ for a normal value of 1500 elements/mm^3^. After resection of the mass, pathology confirmed the presence of diffuse epithelioid-gigantocellular granulomatous inflammation with caseous necrosis. The diagnosis was confirmed by the detection of *M. tuberculosis* using GeneXpert. The patient was given anti-tuberculosis treatment for nine months according to the 2RHZE/7RH regime, that is, two months of rifampin/isoniazid/pyrazinamide/ethambutol followed by seven months of rifampicin/isoniazid.

## Discussion

Tuberculosis remains an endemic disease in Morocco, but parietal localization remains a rare extrapulmonary form. In fact, this represents only 0.1% of tuberculosis locations [1] and does not display typical clinical signs of tuberculosis [2]. Parietal tuberculosis is well-known to touch the ribs and intercostal spaces due to hematogenous spread or direct transcutaneous inoculation.

Clinically, it presents in the form of a cold abscess, pseudo-tumoral mass, or renitent cystic mass, painless without surrounding inflammatory signs. Generally, it has a single location with predilection along the ribs and in the parasternal region. The multiplicity of lesions should prompt immunosuppression [3].

Consequently, this case reminds us that the diagnosis of tuberculosis must be considered when lymphopenia is present, in terms of the endemic context, and when the parietal mass is associated with micronodules, even if the patients are immunocompetent [4].

Surgical excision of the mass allows a tuberculosis diagnosis to be made, but it must be associated with medical treatment to avoid any local or distant recurrences [5]. Medical treatment alone is often insufficient and must be associated with surgical excision or debridement [6].

## Conclusions

Chest wall tuberculosis is a very rare form of tuberculosis that can present as a cold abscess or pseudo-tumoral mass. The diagnosis must be considered in the face of numerous clinical and radiological arguments. Its treatment is based on antibacillary chemotherapy with an average duration of six to nine months and surgical excision. This case reminds us that the diagnosis of tuberculosis must be considered given the endemic context, even in immunocompetent patients.
